# Intradiverticular Ampulla of Vater: Personal Experience at ERCP

**DOI:** 10.1155/2013/102571

**Published:** 2013-07-01

**Authors:** Girolamo Geraci, Giuseppe Modica, Carmelo Sciumè, Antonio Sciuto

**Affiliations:** Section of General and Thoracic Surgery, University of Palermo, Via Liborio Giuffrè 5, 90127 Palermo, Italy

## Abstract

*Introduction*. Conflicting results have been reported about the true impact of intradiverticula ampulla (IA) on the technical success and complication rate of endoscopic retrograde cholangiopancreatography (ERCP). *Patients*. A total of 500 patients who underwent ERCP were divided into two groups according to the presence (group A, 81 patients) or absence (group B, 419 patients) of IA. Success rate, difficulty at cannulation, findings at ERCP, and procedure-related complications were retrospectively reviewed. *Results*. Successful cannulation was achieved in 100% of group A patients compared to 98% of group B patients (*P* = ns). There was a significant difference in the type of cannulation that was routinary in group B (*P* < 0.05), while requiring guidewire in group A (*P* < 0.05). Cholangitis (*P* < 0.05), microstones (*P* < 0.01), dilated common bile duct without stones (*P* < 0.01), stone recurrence (*P* < 0.01), and transient postprocedure hyperamylasemia (*P* < 0.01) were more frequently observed in group A. There was no significant difference in complication rate between both groups. *Conclusions*. The finding of an IA at ERCP should not be considered a predictor for failed cannulation. IA is associated with post-ERCP transient hyperamylasemia and is a risk factor for biliary stone disease and its recurrence.

## 1. Introduction

Intradiverticular ampulla (IA) or periampullary duodenal diverticula (PDD) are found in 9% to 32% of patients undergoing endoscopic retrograde cholangiopancreatography (ERCP). One of the myths of operative biliary endoscopy is that IA makes deep biliary cannulation difficult or even impossible [1].

The aim of our study is to investigate the impact of PDD on technical success and complications of an ERCP.

## 2. Materials and Methods

A total of 551 consecutive ERCPs were performed from January 2008 to December 2012 at our surgical endoscopy unit. Fifty-one patients were excluded from the study because of prior endoscopic sphincterotomy (*n* = 12) or placement of stent (*n* = 20) or undetectable papilla (*n* = 19) after a thorough examination of the second duodenal portion. None of the excluded patients had PDD. As a result, this study includes 500 patients, who were divided into two groups according to the presence or absence of PDD. There were 218 males and 282 females with a mean age of 59.2 years (range, 18 to 89 years). Indications for ERCP included gallstone pancreatitis, common bile duct (CBD) stones, cholangitis, neoplasms (CBD and pancreas), and pancreas divisum. Data were collected from a prospectively maintained database.

All the procedures were performed by the same operator, using Olympus TJF 145 side-view endoscopes. All patients received a combination of midazolam and fentanyl, on escalating dosing according to the needs for conscious sedation. Supplemental oxygen was given transnasally during the entire procedure. Biliary cannulation was attempted by using a standard three-lumen sphincterotome after the intravenous administration of hyoscine butylbromide 20 mg. Cannulation was considered successful when the sphincterotome was inserted deeply into the CBD and a cholangiogram was obtained. When biliary cannulation was not achieved by standard sphincterotome, we used hydrophilic guidewire or needle-knife precut papillotomy with or without a pancreatic stent. Sphincterotomy was then completed with an endocut mode. Difficulty at cannulation was graded as follows: grade I, easy deep cannulation with routinary methods; grade II, requirement of guidewire or special sphincterotome to achieve cannulation; grade III, difficult cannulation that requires special techniques and skills as needle-knife sphincterotomy; grade IV, impossibility of deep cannulation [1]. 

IA related to major papilla was classified according to Boix into the following: type I, papilla located inside the diverticulum (Ia “up,” Ib “left,” Ic “down,” and Id “right”); type II, papilla located in the margin of the diverticulum (IIa “apical left margin,” IIb “apical right margin,” IIc “center left or right margin,” and IId “between two diverticula”); type III, papilla located near of the diverticulum [1]. 

Success rate, difficulty at cannulation, findings at ERCP, and procedure-related complications in patients with IA (group A) were compared to those of patients with normal duodenum (group B). Demographic features as well as indications for ERCP were also evaluated. Statistical analysis was done by Student's *t*-test for continuous variables and by *χ* ^2^ test or Fisher's exact test for discrete variables. A *P* value <0.05 was considered significant.

## 3. Results

IA was observed in 81 of 551 patients, accounting for a prevalence rate of 14.7%. The papilla was undetectable at ERCP in none of the patients with PDD compared to 19 of 470 patients (4.04%) without diverticula.

Mean age was significantly higher (*P* < 0.05) in group A (69.5 years) than in group B (49.7 years). Demographic and clinical features of the patients referred for ERCP are compared in Table 1. Cholangitis was significantly more common in group A (38.3%) than in group B (22.9%), while a lower prevalence of pancreatic or CBD neoplasms was found among patient with IA (9.9% versus 19.5%, *P* < 0.05).

**Figure 1:**
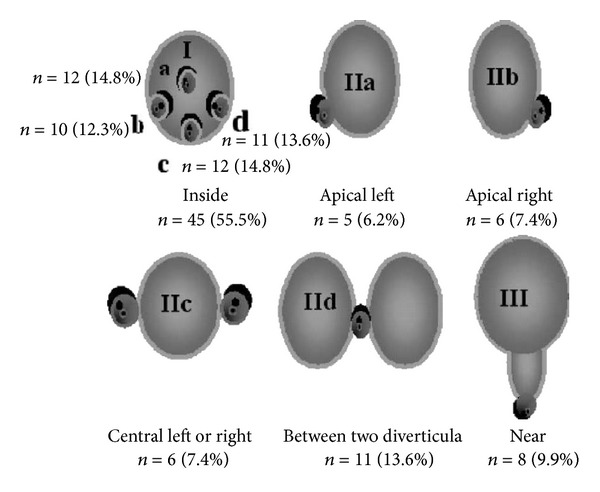
Incidence of the different types of PDD according to the position of the major papilla (modified from [1]).

Incidence of PDD is detailed in Figure 1. The most common diverticula were type I (56%), followed by type II (35%) and type III (9%).

The ampulla was detected in all patients with PDD. Deep biliary cannulation was achieved in 100% of patients with IA compared to 98% of patients without IA (*P* = ns). Easy cannulation with standard methods was achieved in 84.2% of group B patients compared to 7.4% of group A (*P* < 0.05); guidewire or special sphincterotome was required to achieve cannulation in 88.9% of group A patients compared to 12.6% of group B (*P* < 0.05). No statistically significant difference in the use of needle-knife precut papillotomy with or without a pancreatic stent was observed between both groups.

At ERCP, biliary sludge with microstones and dilated CBD in absence of stones were more frequently discovered in group A (*P* < 0.01), while pancreatic cancer and cholangiographic abnormalities were prevalent in group B (*P* < 0.01). Moreover, patients with IA had higher incidence of postoperative transient hyperamylasemia as well as CBD stones recurrence at 1-year followup (*P* < 0.01). Success rate, difficulty at cannulation, ERCP findings, procedure-related complications, and CBD stone recurrence are summarized in Table 2.

## 4. Discussion

PDD (or IA) were first described by Chomel in 1710. They are extraluminal outpouchings of the duodenum adjacent to or containing the ampulla of Vater. PDD are true acquired pulsion diverticula, which involve all layers of the duodenal wall and occur because of abnormal motility. They are usually asymptomatic, but they can also be associated with significant morbidity and, rarely, mortality [1, 2]. 

The true prevalence of PDD in the general population is uncertain due to the diagnostic accuracy of various methods. Prevalence rates on radiographic studies are as high as 5-6%, while those at necroscopic investigations range from 5% to 19.4%. Prevalence rates from 4–9% to 25–32.8% have been reported at esophagogastroduodenoscopy, the average being from 10% to 20% [1, 3]. The higher rates reported at ERCP, ranging from 6.8% to 54.9%, could be explained by a better visualization of diverticula with lateral view duodenoscope [3]. PDD are frequently discovered in elderly patients, and it is generally accepted that their incidence increases with age [1–4]. In our study, the prevalence of PDD was 16.2% and the mean age of patients with diverticula was 69.5 years.

Panteris et al. found a significantly higher prevalence of undetectable papilla in patients with PDD, probably related to the possible location of the papilla inside a diverticulum [3]. This was not confirmed in our study, where none of the patients with diverticulum had undetectable papilla.

Most of the studies conducted to evaluate the impact of PDD on technical issues as well as difficulty and potential complications of an ERCP are inconclusive as to whether diverticula are really only a benign bystander or a threat to successful, easy, and safe cannulation and sphincterotomy [3]. Although successful cannulation rate in patients with PDD ranges from 61% to 95.4%, this was found to be significantly lower compared to that of patients without PDD in 4 of 7 studies reported in the literature [4–7]. In the remaining 3 studies [1, 8, 9], no statistically significant difference was observed. Likewise, difficulty at cannulation was addressed in 3 studies [1, 6, 7]: in 2 of them [6, 7], the results were significantly toward a more difficult cannulation in patients with diverticula, whereas no difference was found in the third study [1]. Difficulty was assessed by the duration (>15 minutes) until cannulation [6], the number of attempts (>10) for cannulation, or the method used to achieve cannulation [1]. 

The presence of PDD is thought to make ERCP a technically demanding procedure, with relatively low success rates. However, this belief is based on results from older studies [2]. Cannulation of papilla situated deep in the large diverticula can be difficult and time consuming and requires more expertise [10]. In our experience, the presence of PDD did not compromise the success rate, although it required the use of guidewire to achieve cannulation (grade II difficulty according to Boix) in most cases. Traditional landmarks for performing sphincterotomy in this setting could be obscured when the papilla is located at the edge of the diverticulum and the biliary intraduodenal portion is not visible. In this case, sphincterotomy should be performed over a guidewire, with only few millimeters of the cutting wire inside the papilla; directing the cut toward the base of the diverticulum should be strictly avoided. Conversely, when the papilla is located in the middle of a diverticulum, the intraduodenal portion is outlined and the landmarks are usually more obvious than in normal anatomy [11].

Association between PDD and ERCP-related complications, as bleeding and duodenal perforation, is controversial [1, 3, 6, 10]. This study supports the majority of published data that PDD do not increase the risk for such complications. Only transient hyperamylasemia without clinical significance had a higher incidence among patients with diverticula. Perforation and bleeding were even less common in this group.

Relationship between PDD and symptoms or clinical findings is not clear [1]. The only scientific evidence, dated back to 1980, is that PDD dispose primarily to gallstone disease [6, 9, 12–14], because of an ascending infection with glucuronidase producing bacteria, related to biliary stasis [15, 16]; *Escherichia coli*, *Streptococcus faecalis*, *Proteus* and *Klebsiella* spp., and anaerobes are the most common organisms isolated [2]. In our series, biliary sludge and microstones were more frequent in patients with diverticula but not CBD stone >10 mm. Moreover, 37.1% of patients with PDD had dilated CBD in absence of stones. In such cases, biliary symptoms could be related to the presence of diverticula, with subsequent biliary stasis and bile duct dilation on US or MRI [3]; in group B, 5% of patients had CBD dilated without stones at ERCP; they were affected by sphincter of Oddi dysfunction or papillodditis, presenting as gallstones pancreatitis or cholangitis.

Finally, in this study a higher CBD stone recurrence was associated with PDD. This can be explained by the decision of the endoscopist to perform small sphincterotomies, especially when the papilla was in an awkward position with respect to the diverticulum, for fear of causing a perforation [3].

## 5. Conclusions

IA is not an uncommon incidental finding in Sicilian adult patients undergoing ERCP. In our experience, the presence of diverticula does not either affect the success rate of ERCP or the incidence of procedure-related complications. Cannulation can be achieved by using a guidewire in most cases. PDD represent a risk factor for choledocholithiasis and its recurrence.

## Figures and Tables

**Table 1:** Demographics and clinical presentation of the patients referred for ERCP.

	Group A *n* = 81	Group B *n* = 419	*P*
Mean age (yrs)	69.5	49.7	<0.05
Range of age (yrs)	54–89	18–75	na
M/F ratio	36/45	182/237	na
Indications			
Gallstone pancreatitis	30/81 (37%)	138/419 (32.9%)	0.51 (ns)
CBD stones	53/81 (65.4%)	257/419 (61.3%)	0.48 (ns)
Cholangitis	31/81 (38.3%)	96/419 (22.9%)	<0.05
Neoplasms (CBD and pancreas)	8/81 (9.9%)	82/419 (19.5%)	<0.05
Pancreas divisum	—	1/419 (0.2%)	0.85 (ns)

ns: not significative; na: not applicable; CBD: common bile duct.

**Table 2:** ERCP results.

	Group A *n* = 81	Group B *n* = 419	*P*
Successful cannulation	81/81 (100%)	412/419 (98%)	0.2 (ns)
Difficult cannulation (Boix scale)			
Grade I	6/81 (7.4%)	353/419 (84.2%)	<0.05
Grade II	72/81 (88.9%)	53/419 (12.6%)	<0.05
Grade III	3/81 (3.7%)	6/419 (1.4%)	0.16 (ns)
Grade IV	0	7/419 (1.7%)	0.24 (ns)
CBD stones > 10 mm	13/81 (16.1%)	61/419 (14.5%)	0.72 (ns)
Biliary sludge and microstones	27/81 (33.3%)	68/419 (16.2%)	<0.005
Ampulloma/ampullary cancer	0	21/419 (5.0%)	0.07 (ns)
Biliary hilar cancer (Klatskin)	3/81 (3.7%)	31/419 (7.4%)	0.22 (ns)
Pancreatic cancer	0	48/419 (11.4%)	<0.005
Cholangiographic abnormalities (stones, strictures, dilation)	51/81 (62.9%)	398/419 (95%)	<0.005
Dilated CBD without stones	30/81 (37.1%)	21/419 (5.0%)	<0.005
Other (liver metastasis, hemobilia)	2/81 (5.5%)	6/419 (1.4%)	0.49 (ns)
Complications			
Clinical bleeding	1/81 (1.2%)	15/419 (3.6%)	0.27 (ns)
Perforation	0	2/419 (0.5%)	0.53 (ns)
Pancreatitis	2/81 (2.4%)	12/419 (2.9%)	0.84 (ns)
Cholecystitis	0	0	na
Death (ERCP related)	0	0	na
Immediate bleeding	1/81 (1.2%)	14/419 (3.3%)	0.31 (ns)
Hyperamylasemia	52/81 (64.2%)	106/419 (25.3%)	<0.005
CBD stone recurrence at followup	53/81 (65.4%)	38/419 (9.1%)	<0.005

ns: not significative; na: not applicable; CBD: common bile duct.
